# Emphysematous Pyelonephritis Revealing Underlying Renal Tuberculosis: A Rare and Challenging Association

**DOI:** 10.7759/cureus.100462

**Published:** 2025-12-30

**Authors:** Anass El Alaoui, Abdessamade Motaouakil, Mohammed Ramdani, Anouar El Moudane, Ali Barki

**Affiliations:** 1 Department of Urology, Mohammed VI University Medical Center, Oujda, MAR; 2 Department of Urology, Faculty of Medicine and Pharmacy, Mohammed the First University, Oujda, MAR

**Keywords:** clinical case report, diabetes mellitus type 2, emphysematous pyelonephritis (epn), mycobacterium tuberculosis, renal tuberculosis

## Abstract

Emphysematous pyelonephritis (EPN) is a severe, necrotising infection of the renal parenchyma and surrounding tissues, most often accompanied by diabetes mellitus and Gram-negative bacilli. Tuberculosis superinfection is very rare, with only a few cases reported in the literature.

We present the case of a 61-year-old woman with multiple comorbidities, in whom emphysematous pyelonephritis (EPN) was primarily due to typical bacterial pathogens and subsequently complicated by a superinfection with *Mycobacterium tuberculosis*, highlighting the importance of recognizing this unusual secondary infection to avoid unnecessary nephrectomy.

## Introduction

Emphysematous pyelonephritis (EPN) is a potentially life-threatening infection characterized by the presence of gas in the renal parenchyma, the excretory tract, or the perirenal tissues [1].

It is strongly associated with uncontrolled diabetes mellitus and is usually caused by microorganisms such as Escherichia coli or Klebsiella pneumoniae [1]. Tuberculous superinfection of EPN is extremely rare, with only a few cases described worldwide [2].

We report an exceptional case of EPN primarily caused by common bacterial pathogens in a diabetic patient, which was subsequently complicated by a superinfection with Mycobacterium tuberculosis, highlighting the diagnostic challenges and therapeutic implications.

## Case presentation

A 61-year-old woman with a medical history of chronic rheumatic disease with long-term corticosteroid therapy (Prednisone 10 mg/day) presented with right-sided flank pain and fever. She had recently been diagnosed with type 2 diabetes mellitus. She reported recent exposure to tuberculosis in her family home, as her husband had died of respiratory failure related to pulmonary tuberculosis.

When admitted, the patient had a fever and tenderness in her right lumbar region. Laboratory tests revealed a severe inflammatory syndrome (Table 1).

**Table 1:** Laboratory findings at admission

Parameter	Result	Unit	Reference / Comment
C-reactive protein (CRP)	483	mg/L	Markedly elevated, indicating severe inflammatory response
Leukocyte count	14,300	/µL	Leukocytosis
Procalcitonin (PCT)	20.82	ng/mL	Highly elevated, consistent with severe bacterial infection or sepsis
Serum creatinine	35	µmol/L	Slightly decreased
Urine culture	Klebsiella pneumoniae (multiresistant)	—	Confirmed urinary tract infection with multidrug-resistant organism

**Figure 1:**
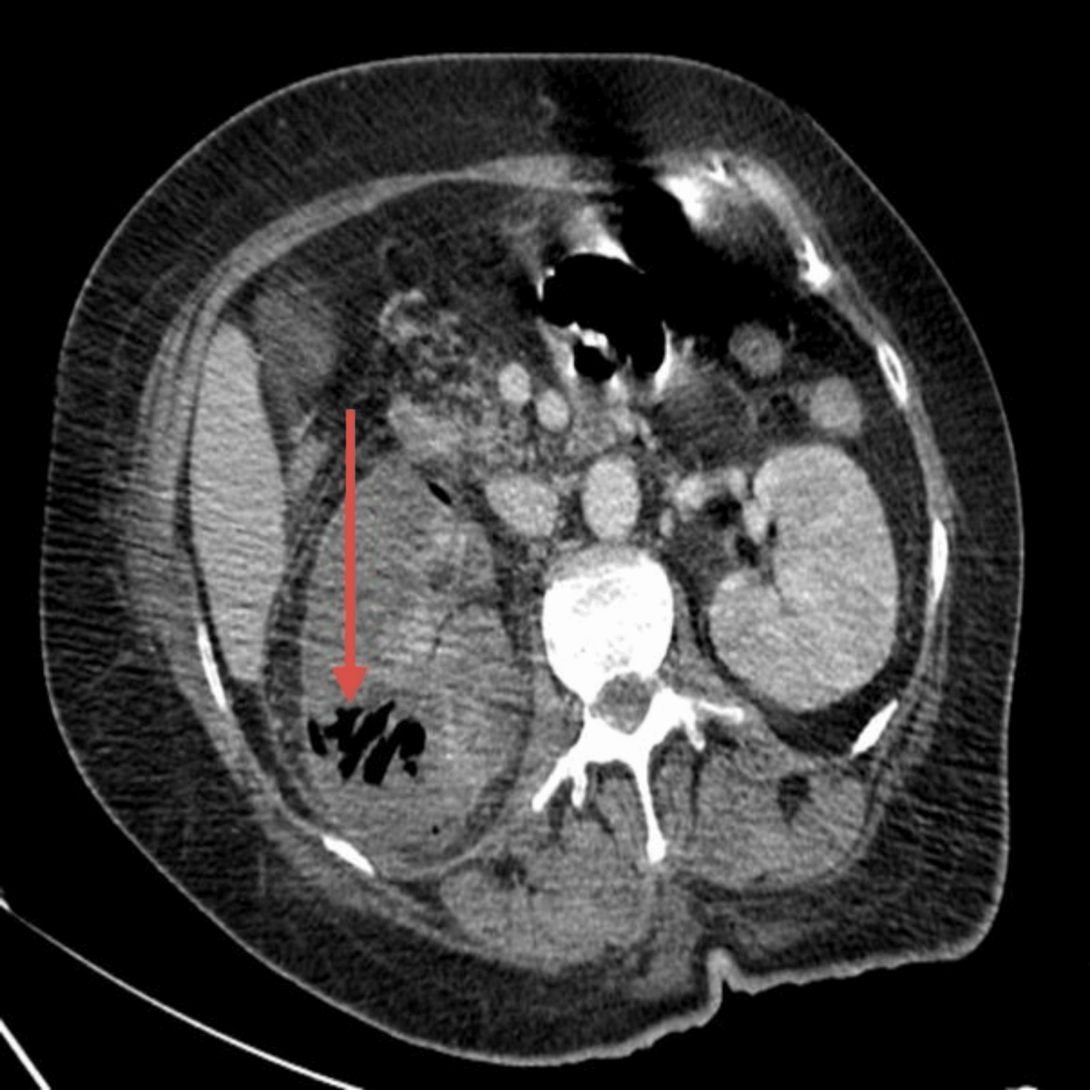
Axial CT scan showing gas within the right renal parenchyma.

**Figure 2:**
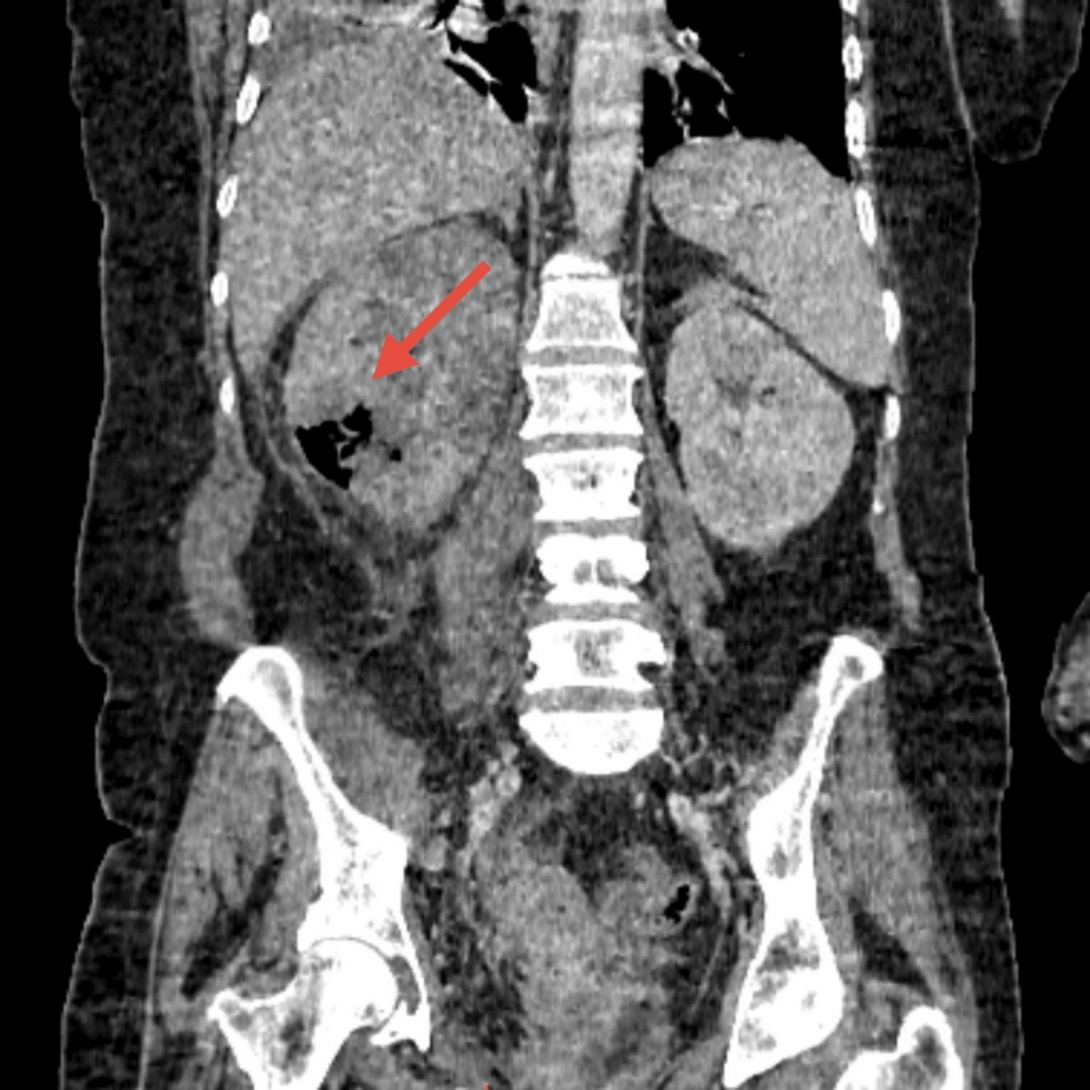
Coronal CT scan showing gas within the right renal parenchyma.

An abdominal-pelvic computed tomography (CT) scan with contrast injection revealed right emphysematous pyelonephritis with gas present in the renal parenchyma and collecting system (Figures 1-2). According to the Huang and Tseng classification, the present case was classified as Class 2 emphysematous pyelonephritis [1].

The patient was placed on empirical intravenous antibiotics (ceftriaxone plus a single dose of amikacin) and underwent double J stent placement with pyelic sampling for microbiological analysis, including a tuberculosis test.

Despite adequate antibiotic treatment and urinary drainage, his clinical and biological condition remained unfavourable, with persistent fever and elevated inflammatory markers. Direct examination for acid-fast bacilli and blood cultures were negative. Anti-tuberculosis treatment was not initiated at this stage due to a lack of evidence.

The definitive diagnosis of a tuberculous superinfection was made when the culture of the pyelic sample revealed the presence of Mycobacterium tuberculosis. Anti-tuberculosis therapy (isoniazid, rifampicin, pyrazinamide, and ethambutol) was added to the ongoing antibiotic treatment, leading to a marked improvement in the patient's clinical condition, with resolution of fever and normalization of inflammatory markers.

## Discussion

Emphysematous pyelonephritis (EPN) is most commonly caused by Gram-negative bacteria, particularly Escherichia coli and Klebsiella pneumoniae [1]. Tuberculosis, when present, usually represents a superinfection rather than a primary etiology. Only a few cases of tuberculous superinfection in EPN have been reported to date [2-3].

In this case, several risk factors were present, including uncontrolled diabetes mellitus, chronic corticosteroid treatment, and recent exposure to tuberculosis. It is important to note that the initial urine culture revealed the presence of Klebsiella pneumoniae, which may have delayed the diagnosis. However, the lack of response to conventional antibiotics led to further evaluation, which eventually confirmed the presence of M. tuberculosis.

Renal tuberculosis may promote emphysematous infection indirectly by inducing chronic inflammation, tissue destruction, local ischemia, and impaired host immune response, thereby facilitating secondary infection by gas-forming organisms.

This case underscores the importance of considering tuberculosis superinfection in patients with EPN who show poor response to appropriate antibiotic therapy, particularly in endemic regions or patients with a history of exposure. Early collection of pyelic samples for mycobacterial culture is crucial, as direct microscopy may be negative.

Radiological imaging plays a key role in the diagnosis and management of EPN. Computed tomography (CT) is the modality of choice, as it allows for accurate identification of gas in the renal parenchyma, its extension to the perinephric tissues, and classification of the pathology, which correlates with prognosis and guides therapeutic decisions [1-4]. In our case, CT confirmed the diagnosis of class 2 right-sided EPN, according to the Huang and Tseng classification [1], guiding the urgent placement of a double J stent for urinary drainage.

The management of EPN has evolved considerably and is guided by disease severity and CT scan classification. Historically, emergency nephrectomy was the standard approach, but conservative management combining antibiotics and percutaneous or endoscopic drainage is increasingly preferred, particularly in less severe cases [5]. Our patient initially underwent conservative treatment with antibiotics and placement of a ureteral stent. However, the lack of response necessitated further tests, which ultimately revealed the presence of Mycobacterium tuberculosis. After starting anti-tuberculosis treatment in addition to ongoing antibacterial therapy, the patient showed rapid clinical improvement.

## Conclusions

Tuberculosis should be considered in patients with emphysematous pyelonephritis who show poor response to appropriate antibiotic therapy, particularly in endemic regions or in immunocompromised individuals. Early mycobacterial testing in antibiotic-resistant cases may prevent delayed diagnosis, disease progression, and unnecessary surgical interventions such as nephrectomy.
